# Subjective right ventricle assessment by echo qualified intensive care specialists: assessing agreement with objective measures

**DOI:** 10.1186/s13054-019-2375-z

**Published:** 2019-03-07

**Authors:** Sam Orde, Michel Slama, Konstantin Yastrebov, Anthony Mclean, Stephen Huang, on behalf of the College of Intensive Care Medicine of Australia and New Zealand [CICM] Ultrasound Special Interest Group [USIG], Andras Nyikovics, Deirdre Murphy, Geoff Gordon, Lewis Campbell, Li Tan, Mate Rudas, Michael Farqharson, Nicola Stanley, Pierre Janin, Russell Laver, Soumya Ray, Vinodh Nanjayya, Ramsy D’Souza, Paul Healey, Bradley Wibrow, Florian Pracher, Kyle Brooks, David Clancy, Thomas Hamp, Sebastian Knudsen, Joe McCaffrey, Godfrey Lo, Sutrisno Gunawan, Arvind Rajamani, Marek Nalos, Ian Seppelt, Alison Main, Yang Yang, Andrew Hilton, Pranesh Jogia, Jude Bhathan, John Evans, Priya Nair, Lyndal Russel, Mani Gopal, Matthew Anstey, Josh Ihle, Alvin Teo, Ben Cheung, Matt Brian, Richard Strickland, Cartan Costello, Peter McCanny, Siew Wai, Irma Bilgram

**Affiliations:** 1 Nepean Hospital, Sydney, NSW Australia; 2 Medical ICU, Amiens University Hospital, Amiens, France; 3 St George Hospital, Sydney, NSW Australia

**Keywords:** Right ventricle, Subjective assessment, Speckle tracking, Critically ill, ICU

## Abstract

**Background:**

Right ventricle (RV) size and function assessment by echocardiography (echo) is a standard tool in the ICU. Frequently subjective assessment is performed, and guidelines suggest its utility in adequately trained clinicians. We aimed to compare subjective (visual) assessment of RV size and function by ICU physicians, with advanced qualifications in echocardiography, vs objective measurements.

**Methods:**

ICU specialists with a qualification in advanced echocardiography reviewed 2D echo clips from critically ill patients on mechanical ventilation with PaO_2_:FiO_2_ < 300. Subjective assessments of RV size and function were made independently using a three-class categorical scale. Agreement (*B*-score) and bias (*p* value) were analysed using objective echo measurements. RV size assessment included RV end-diastolic area (EDA) and diameters. RV function assessment included fractional area change, S′, TAPSE and RV free wall strain. Binary and ordinal analysis was performed.

**Results:**

Fifty-two clinicians reviewed 2D images from 80 patients. Fair agreement was seen with objective measures vs binary assessment of RV size (RV EDA 0.26 [*p* < 0.001], RV dimensions 0.29 [*p* = 0.06]) and function (RV free wall strain 0.27 [*p* < 0.001], TAPSE 0.27 [*p* < 0.001], S′ 0.29 [*p* < 0.001], FAC 0.31 [*p* = 0.16]). However, ordinal data analysis showed poor agreement with RV dimensions (0.11 [*p* = 0.06]) and RV free wall strain (0.14 [*p* = 0.16]). If one-step disagreement was allowed, agreement was good (RV dimensions 0.6 [*p* = 0.06], RV free wall strain 0.6 [*p* = 0.16]). Significant overestimation of severity of abnormalities was seen with subjective assessment vs RV EDA, TAPSE, S′ and fractional area change.

**Conclusion:**

Subjective (visual) assessment of RV size and function, by ICU specialists trained in advanced echo, can be fairly reliable for the initial exclusion of significant RV pathology. It seems prudent to avoid subjective RV assessment in isolation.

**Electronic supplementary material:**

The online version of this article (10.1186/s13054-019-2375-z) contains supplementary material, which is available to authorized users.

## Background

The importance of the right ventricle (RV) in the management of critically ill patients is increasingly recognised [1]. RV dilation and dysfunction are common in the ICU and are associated with worse outcomes in disease states such as ARDS, septic shock and heart failure [2–5]. Echocardiography (echo) plays a crucial role in RV assessment for both diagnosis and monitoring and is an essential tool for the management of these patients in the ICU [1]. The use of echo in the critical care environment is increasing around the world [6] as is research in this area [7]. Echo is well known to be user dependent both in image acquisition and analysis [8]. Leading national echocardiography society guidelines suggest to examine the heart from multiple acoustic windows with overall assessment to be based on subjective assessment in addition to quantitative parameters [9]. Subjective assessment (‘eyeballing’) of the RV is rapid and remains a common method used clinically [10] especially in the ICU. However, the accuracy, inter-observer and intra-observer concordance is not well described, particularly for critical care physicians.

The RV is not always easy to image with ultrasound. It has a crescentic shape, wrapped around the left ventricle with a retrosternal position. There are numerous methods available to measure RV size and function, yet the parameter that is the most accurate in the critically ill is controversial. With regard to RV size, basal, mid and longitudinal dimensions (in the apical four-chamber view) have been validated as well as the end-diastolic area (again from the apical four-chamber view) [9]. However, it is worth noting that these reference values are based on published data obtained from normal adults without any history of heart or pulmonary disease. Multiple measures are also used for the assessment of RV function, including TAPSE (tricuspid annular plane systolic excursion), fractional area change (FAC) and S′ (systolic velocity on tissue Doppler imaging) and the relatively novel and sensitive parameter RVfwS (assessed by speckle tracking).

We sought to compare subjective RV size and function assessment by intensive care specialists with qualifications in critical care echo (CCE) vs quantitative RV echo measurements (using RVfwS as the primary reference method) in critically ill patients. We hypothesised that there would be fair to good agreement.

## Methods

Fifty-two currently practising intensive care specialists with a qualification in advanced echocardiography were invited to review, offline and in a blinded fashion, a selection of 2D echo clips of 80 critically ill patients. Participants were asked to subjectively estimate RV size and function based on the following categories: normal, mild/moderate or severely abnormal (see Additional file 1: Appendix 1 for data sheet), which were deemed clinically relevant by the authors. A presentation was provided to the clinicians consisting of 80 slides, one per patient, with three to five video clips on each slide (depending on echo windows available) (see Additional file 2: Appendix 2 for example). All images were obtained from patients at a single-centre, tertiary hospital. Participants were instructed to review in their own time. There was no clinical data provided for individual patients, only the general inclusion criteria.


**Additional file 2: Appendix 2.** Example of 2D echo images to be reviewed in order to assess subjectively right ventricle size and function. (M4V 3986 kb)


An intensive care specialist was defined as a clinician who is a Fellow of the College of Intensive Care Medicine (CICM) of Australia and New Zealand, or who had passed their final exams and were in their ‘fellowship’ (final) year of training. Advanced and expert levels of training in CCE were defined in accordance with recommendations on levels of training in CCE by the CICM Ultrasound Special Interest Group (USIG) [11]. The definition of the expert level of training included CCE experience in excess of 7 years of practice; thus, this period was used in sub-group analysis.

The project was approved by the Nepean Blue Mountains Local Health District (LNR/13/NEPEAN/154). Imaging of patients was performed after written consent being provided prospectively by the authorised representative (next of kin) or retrospectively by the patient (deemed reasonable given echo being considered standard of care in our unit and the non-invasive nature of the imaging).

### Patients

Inclusion criteria for critically ill patients imaged included adult (> 18 years), mechanically ventilated (pressure support or mandatory ventilation) with a ‘significant’ ventilation-perfusion (VQ) mismatch defined as a PaO2:FiO2 ratio < 300. Non-consecutive patients were imaged within 24 h of admission when S.O. was able to review and consent patients. Exclusion criteria included pregnant women, congenital heart disease, previous cardiac surgery, patients undergoing palliative treatment or having inadequate echo imaging to be able to perform STE and assess RVfwS.

### Echocardiography

Transthoracic echocardiography images were acquired by S.O. or experienced sonographers (all highly trained and fully qualified in comprehensive critical care echo) using either a Vivid 7 machine (GE Medical systems, Chicago, USA) using a M4S probe, or a Siemens SC2000 using a 4V1c transducer (Siemens Healthineers, Erlangen, Germany). A standard comprehensive study was performed which included conventional 2D (or B-mode images) as per current American Society of Echocardiography (ASE) guidelines [9]. In addition, non-standard ‘RV centric’ views optimised for speckle tracking were obtained: three cardiac cycles in sinus rhythm, five in atrial fibrillation, reduced depth and width, frame rate > 50 fps, single focal point and ensuring the RV free wall endocardium was seen throughout the cardiac cycle.

RV dilation was defined by RV long axis greater than 83 mm, RV mid-diameter greater than 35 mm and RV basal diameter greater than 42 mm [9] with categorical data based on two dimensions being abnormal defining mild/moderate dilation and all three dimensions being abnormal defining severe. Categorical RV end-diastolic area (RV EDA) definitions included < 29cm^2^ being normal, 29–38 cm^2^ being mild/moderately dilated and greater than 38cm^2^ being severely dilated [12]. RV function by STE was defined as RVfwS more negative than − 21% being normal, between − 13 and − 21% being mild/moderately abnormal and less negative than − 13% being severely abnormal. These values have been used in previous studies in critically ill patients [3], normal subjects [13] and pulmonary hypertension patients [14]. TAPSE categorical data was defined as normal greater than 16 mm [9], mild/moderate dysfunction 10–16 mm and severe dysfunction less than 10 mm [15]. Fractional area change and S′ were assessed in a binary fashion as per ASE guidelines: cut-off of 35% and 9.5 cm/s^2^ respectively as no published categorical data for severity was found.

### Speckle-tracking echocardiography (STE)

RVfwS was assessed by speckle-tracking echocardiography (STE): a relatively novel method increasingly used in critical care echo, however, primarily from a research perspective at this stage. STE is a post-processing software (i.e. a computer program analyses the echo images once they are stored) that tracks the movement of the speckles that make up the myocardium (known as ‘kernels’) throughout the cardiac cycle [16] to determine the ‘strain’ parameter. Systolic strain values are negative, indicating degree of deformation. The more negative a value, the greater the degree of deformation and the greater the systolic function. Although RV systolic function can be assessed by both free wall and ventricular septal strain analysis, using only strain of the free wall (RVfwS) is preferred and has been shown to be sensitive [17], have superior prognostic characteristics over conventional parameters in pulmonary hypertension cohorts [14] and be feasible in the critically ill [18].

The 2D digital clip (3 cycles for sinus rhythm, 5 for atrial fibrillation) RV-centric, apical four-chamber views were transferred to a Tomtec system for STE analysis (TomTec Imaging, Edisonstrasse, Germany). STE analysis was performed by S.O (experienced in this form of evaluation) in a manner as previously described [3, 19]. RV-centric views were analysed initially, but if they were unable to be used, then apical four-chamber views were assessed. All three RV free wall segments had to be viewed throughout at least one cardiac cycle and tracking sufficient for a patient to be included. If STE was not able to be performed with either of these views, then the patient was excluded. The endocardium was traced manually, at end-systole, starting at the lateral tricuspid annulus with 7–15 points, finishing at the medial annulus. Drift correction was included in tracking. Only the free wall segments were considered as per guidelines [9]. Once accuracy of tracking was assured, the displacement, velocity, strain and strain rate curves were then assessed for smoothness of fit, dyssynchrony, time to peak and correlation. If curves were not acceptable, then tracking was repeated. The same cardiac cycle was chosen for STE values if the patient was in sinus rhythm, but averages were taken if in atrial fibrillation. The digital clips used were analysed three times to ensure consistency of results and the final result chosen was based on the curves with the best smoothness of fit. A 15% random population was assessed for inter-rater (M.S) variability for RVfwS.

### Statistical analysis

A sample size of 50 clinicians reviewing 80 cases was calculated using estimates from previous published data [20] as well as an estimated contingency table based on the presumed spread of normal vs mild/moderate vs severe RV dysfunction that we would see (see Additional file 1: Appendix 3). A power of 80% and significance of 0.05 was considered acceptable for the power calculation. Statistical analysis was performed with JMP Pro version 13 (SAS Institute Inc., Cary, NC, USA). Continuous variables are expressed as mean ± standard deviation (SD) if normally distributed and median with interquartile range (IQR) if not normally distributed. Normality was assessed using the Shapiro-Wilk test. Categorical variables are expressed as the number and percentage with comparisons by Pearson’s chi-square analysis or Fisher’s exact test. *P* values < 0.05 are considered statistically significant. Ordinal categorical analysis (normal vs mild/moderate vs severe) as well as binary analysis (normal vs abnormal) was attempted. Cohen’s kappa and Bangdiwala’s *B*-statistic were used as a measure of concordance. *B*-score interpretation was considered poor less than 0.25, fair 0.25 to 0.49, good 0.5 to 0.74, excellent 0.75 to 0.99 and perfect 1. Bias was assessed by Mann-Whitney-Wilcoxon test for marginal distribution. Agreement charts were used to provide a visual impression of the data (an excellent review article on these charts is suggested [21]). Agreement is determined by the size of the box. Black indicates concordance, grey indicates one adjacent level of agreement (e.g. ‘normal’ chosen when quantifiable result ‘mild/moderate’). The direction of observer bias is reviewed by examining the ‘path of the rectangles’ and how it deviates from the diagonal line (of no bias). Further analysis was done accounting for (a) those with more than 7 years of echo experience (arbitrary level required for ‘significant’ experience in CCE by the CICM USIG), (b) those who felt they practised at a level of a cardiologist and (c) those with DDU vs other qualifications. Intra-rater variability was assessed in eight assessors, who reviewed the same images twice at separate times determined by the assessor. This was analysed by intra-class correlation coefficient.

## Results

**Fig. 1:**
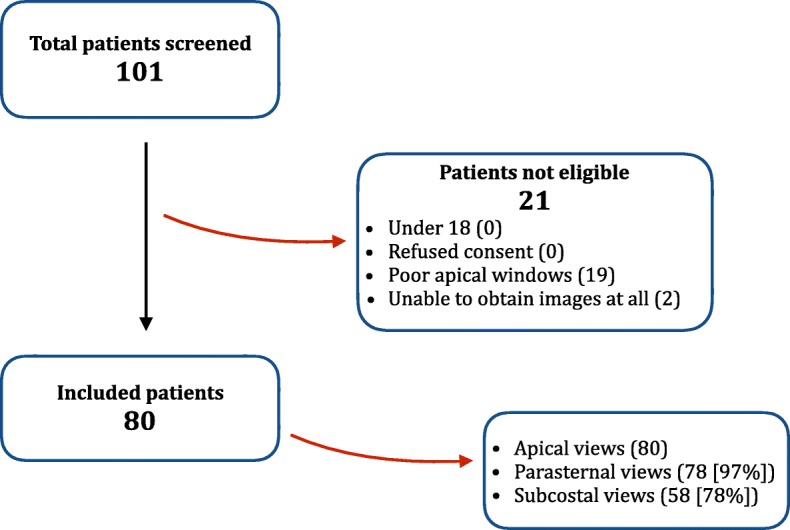
Study flow diagram and echo windows obtained in those included

Fifty-two intensive care specialists reviewed images from the 80 patients (30–120 min reported as time taken to complete study by candidates). An attempt was made to obtain images from apical, parasternal and subcostal windows in all study participants. Screened patients were excluded, when apical views were insufficient for quantification by investigators or when both subcostal and parasternal views were deemed to be of insufficient quality for subjective assessment (see flow diagram in Fig. 1). Feasibility of performing RVfwS in our patient population was 79%. Eighty patients were included: 54% male, median age 68 years (IQR 59 to 73); 91% in sinus rhythm; median P:F ratio 174 (IQR 132 to 208); median PEEP 10 (7 to 12); mean APACHE III 80.5 (± 26); and median time on ventilator 6 days (3 to 9). The right ventricle size and function is displayed in Table 1. Of note, more patients were diagnosed with RV dilation when RV diameters were measured vs end-diastolic area (41% vs 26% respectively [*p* < 0.001]). No significant difference was seen comparing patients classified with abnormal RV function with FAC vs RVfwS (64% and 58% respectively [*p* = 0.8]). However, a significant difference was seen comparing RVfwS vs S′ ([*p* < 0.01]); RVfwS vs TAPSE (*p* < 0.001]) and S′ vs TAPSE (26% and 23% respectively [*p* < 0.001]). RVfwS defined more patients with severe dysfunction vs TAPSE (18% vs 5% respectively [*p* < 0.001]).

**Table 1:** Right ventricle size and function echocardiography data

	Parameter	Value (IQR or ±SD)	% patients with abnormal values (*n*, %)	Categorical data		
				Normal	Mild/moderately abnormal	Severely abnormal
RV size	End-diastolic area (cm^2^)	24.2 (18 to 30)	21 (26%)	59 (74%)	16 (20%)	5 (6%)
	Long axis (mm)	80.8 (± 10)	33 (41%)	27 (34%)	37 (46%)	16 (20%)
	Mid diameter	31.7 (± 7)				
	Basal diameter	40.6 (± 8)				
RV function	Fractional area change (%)	32.5 (± 11)	51 (64%)	–	–	–
	RV S′	11.3 (± 3)	20 (26%)	–	–	–
	TAPSE (mm)	18.9 (16 to 21)	18 (23%)	62 (78%)	14 (18%)	4 (5%)
	RV free wall Strain	− 19.6 (± 6)	46 (58%)	34 (43%)	32 (40%)	14 (18%)

*IQR* interquartile range when describing non-normally distributed data, *SD* standard deviation when describing normally distributed data, *RV* right ventricle, *S′* systolic motion

**Fig. 2:**
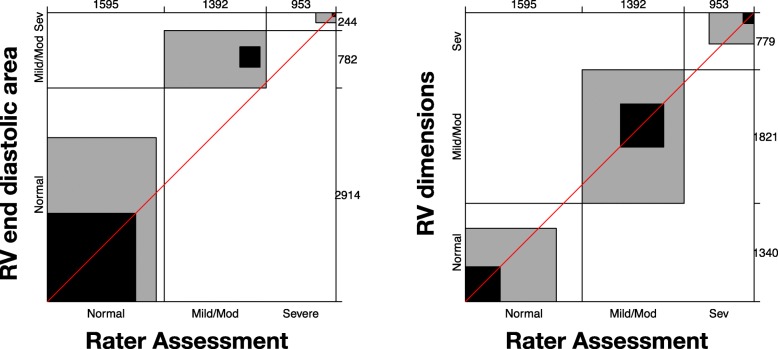
Agreement plot (also called Bangdiwala’s observer agreement chart) of subjective (visual) RV size assessment vs right ventricle end-diastolic area and right ventricle dimensions; please see the ‘Methods’ section or reference [21] for the description of interpretation if needed

**Fig. 3:**
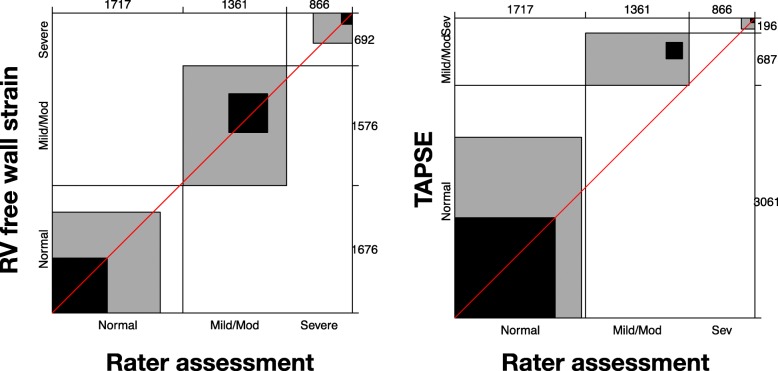
Agreement plot (also called Bangdiwala’s observer agreement chart) of subjective (visual) RV function assessment vs right ventricle free wall strain (assessed by speckle-tracking echocardiography) and TAPSE; please see the ‘Methods’ section or reference [21] for the description of interpretation if needed

The characteristics of the intensive care specialist participants are shown in Table 2. Those participating had considerable clinical (median 4.5 yrs. as a specialist) as well as echo experience (median 7 years). The most common echo qualification held by those participating in the study was the DDU (27 of the 52 involved in the study). Table 3 shows the agreement and bias seen with subjective RV size and function assessment. Agreement was fair for binary (normal vs abnormal) assessment of RV size (subjective vs RV EDA = 0.26 [*p* < 0.001]; vs RV dimensions 0.29 [*p* = 0.06]) as well as for RV function assessment (subjective vs RVfwS = 0.27 [*p* = 0.35]; TAPSE = 0.27 [*p* < 0.001]; vs S′ = 0.29 [*p* < 0.001]; vs FAC = 0.31 [*p* < 0.001]) (see Additional file 1: Appendix 4 for agreement plots based on binary data for subjective RV size and function assessment). Agreement was also fair when assessment was for some ordinal data (normal, mild/moderate, severe) for RV size assessment (subjective vs RV EDA = 0.26 [*p* < 0.001) and RV function assessment (subjective vs TAPSE = 0.28 [*p* < 0.001]). In regard to ordinal data, if one-step disagreement was allowed for (weighted agreement *B*-score), good agreement was seen for RV size assessment (subjective vs RV EDA = 0.62 [*p* < 0.001]; vs RV dimension = 0.59 [*p* = 0.06]) and RV function assessment (subjective vs RVfwS = 0.60 [*p* = 0.16]; TAPSE = 0.65 [*p* < 0.001]) (see Figs. 2 and 3 for agreement plots of subjective RV size and function assessment respectively based on ordinal data). Poor agreement was seen in unweighted ordinal data for subjective assessment vs RV dimension (0.11 [*p* = 0.06]) and RVfwS (0.14 [*p* = 0.16]). Large positive bias (overestimating severity) was seen in the agreement of RV size when assessed by RV EDA (in both ordinal and binary data) and in RV function when assessed by TAPSE and RV S′ in binary data. A small negative (underestimating) bias in assessing RV function was seen when FAC was used as comparator (Additional file 1: Appendix 4).

**Table 2:** Intensive care specialist characteristics

Characteristic	Values (IQR or %)
Years performing CCE (years)	7 (5 to 11)
Approximate number of TTE performed per year (*n*)	100 (63 to 200)
Self-reporting performance of CCE at level of cardiologist (%)	40 (77%)
Years practising as ICU specialist (years)	4.5 (2 to 10)

*IQR* interquartile range when describing non-normally distributed data, *CCE* critical care echocardiography

**Table 3:** Agreement and bias of subjective assessment of RV size and function by Australasian intensive care specialists with advanced and expert level of training in critical care echocardiography

	Data type	Parameter	Agreement (*B*-score)		Bias (*p* value)
			Unweighted	Weighted*	
RV size	Binary	RV end-diastolic area	0.26	–	< 0.001
		RV dimensions	0.29	–	0.06
	Ordinal	RV end-diastolic area	0.26	0.6234	< 0.001
		RV dimensions	0.11	0.5870	0.06
RV function	Binary	RV free wall strain	0.27	–	0.35
		TAPSE	0.27	–	< 0.001
		S′	0.29	–	< 0.001
		Fractional area change	0.31	–	< 0.001
	Ordinal	RV free wall strain	0.14	0.5999	0.16
		TAPSE	0.28	0.6499	< 0.001

Interpretation of *B*-score: poor < 0.25, fair 0.25–0.49, good 0.5–0.75, excellent 0.75 to 0.99 and perfect 1.00

*Weighted agreement allowed for one-step disagreement

**Fig. 4:**
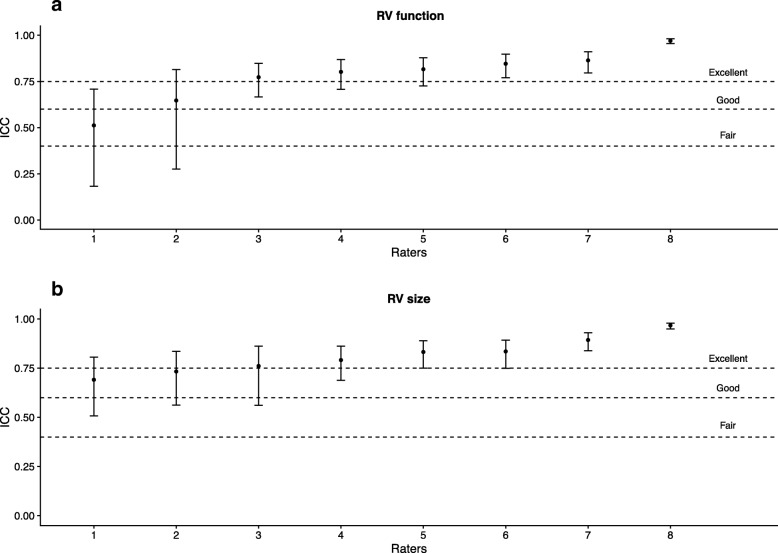
Intra-rater agreement in right ventricle size and function assessment

Using RVfwS and RV dimensions as the reference methods, there was no significant difference seen in agreement when accounting for those with less than more than 7 years of echo experience (see Additional file 1: Appendix 5), whether the participant felt they practised at level of cardiologist (see Additional file 1: Appendix 6) or those with DDU qualification vs others (see Additional file 1: Appendix 7). Fair to excellent correlation was seen in intra-rater agreement of RV size and good to excellent correlation in RV function assessment when they repeated their evaluation (see Fig. 4).

Blinded inter-rater variability for STE analysis was performed on a random 15% of the population (by M.S. and S.O): Bland Altman analysis demonstrated good interrater agreement with mean difference (±standard deviation) 1.1 (± 4.9).

## Discussion

We compared subjective (visual) RV size and function assessment with objective echocardiography measures in 80 critically ill patients by 52 intensive care specialists with qualifications in advanced echocardiography. To our knowledge, this is the largest and most robust analysis of subjective RV assessment vs objective measures performed, particularly in the critically ill. We found fair agreement by clinicians in assessing whether RV size and function was normal vs abnormal compared to conventional echo parameters, as well as the relatively novel and sensitive parameter RVfwS (assessed by speckle tracking). If the categorical data could be assessed in an ordinal manner (normal, mild/moderate, severe), with one-step disagreement allowed, then good agreement was seen. Poor agreement was seen when comparing subjective RV function assessment with RVfwS when unweighted (i.e. concordant) analysis was required. These degrees of agreement remained when accounting for whether or not the clinician had ‘significant’ echocardiography experience (more than 7 years at advanced level), if they felt they practise echo at the level of a cardiologist or for different qualifications.

Subjective assessment was also found to have significantly overestimated severity of RV dilation and dysfunction compared to quantification of RV EDA, TAPSE, S′ and RV fractional area change. Interestingly, no bias was seen when compared with RVfwS, which could potentially be due to STE identifying more abnormalities than conventional parameters. This finding is reaffirmed in other critical care echo studies assessing RVfwS [3]. Raters may be relying more on the change in area and wall motion function in subjective RV function assessment as evident from the reduced bias in results with RVfwS and FAC agreement plots. Although there was only fair agreement, the magnitude and direction were fairly consistent with FAC and RVfwS.

We are not aware of any other studies in CCE assessing subjective vs objective analysis of RV or LV size and function, despite how often this is performed. Cardiology previously addressed LV assessment in a study by Blondheim et al. [22] demonstrating reasonable coefficient of variation and a study by McGowan et al. [23], demonstrating good intra- and inter-observer variability of LV visual quantification by echo. However, the applicability to the ICU population is not known. In the critical care setting, echocardiography is the mainstay of bedside assessment of RV function [1]. Although MRI remains the gold standard for RV assessment outside ICU, it is currently not routinely used in the critically ill. Subjective assessment of the RV is quick and simple and is frequently performed by ICU clinicians and cardiologists. RVfwS has been suggested to be the most sensitive echo parameter to quantitatively describe RV dysfunction [24]. It is now recommended that detailed quantification of the RV should be performed using multi-plane set of images, and include RVfwS [25].

Previous studies assessing the LV using global longitudinal strain demonstrated association with mortality in critically ill patients with sepsis and septic shock, the finding absent using traditional echo quantification of LV [26]. Is it possible that subjective RV assessment might be recognising dysfunction not recognised by the standard quantitative parameters and therefore could be used for prognostications in some groups of critically ill?

The fair agreement between subjective and objective RV assessment identified in our study suggests that visual assessment could be acceptable for initial rapid and crude bedside qualification of RV size and function by critical care physicians with sufficient level of CCE training. Such approach would be useful, for example, in rapid differentiation of shock. In addition, the intra-rater correlation of subjective assessment of RV size and function was very good in more than 80% of the selected sample, suggesting very good consistency of subjective RV rating performed by advanced and expert level CCE users. However, monitoring of RV function during titration of pharmacological and mechanical interventions requires significantly finer level assessment, thus rendering fair level of agreement insufficient. Therefore for the time being, quantitative RV echocardiographic assessment remains the pragmatic reference standard for ICU bedside RV monitoring and detection of subtle changes.

Further studies in this area may consider which RV measure of size and function is actually the most accurate in the critically ill where RV dysfunction appears to be common. It is likely that cardiac MRI studies still may be needed for this. In addition, as multi-centre studies are being performed using echo as the imaging tool, investigating the agreement between critical care physicians performing the studies and acquiring the data would be extremely interesting and valuable.

### Limitations

Our study suffers from several limitations. No clinical context was supplied to the doctors who performed the assessment, only the inclusion criteria to image the patients. This makes analysis different from genuine assessment in the clinical environment which may have an effect. Objective echo analysis was done predominantly by a single user (S.O) including strain analysis, and this may be a factor in terms of feasibility in larger studies. Finally, it is not known which single quantitative parameter describes RV size or function best in the critically ill. TAPSE or S′ represent a surrogate of global RV performance and thus cannot be used in isolation. Arguably, the most sensitive measure of RV function is RVfwS, hence why this was chosen as the primary outcome. However, using RVfwS in this regard limited inclusion of patients who could have suitable imaging performed (RV centric apical view) with a feasibility of 79%, suggesting possible selection bias. The strength of our study is the relatively large sample size, both in number of clinicians as well as patients participating in the analysis.

## Conclusions

Subjective (visual) assessment of the RV size and function can be fairly reliably used in the critical care setting for initial exclusion of significant RV pathology, when performed by intensivists with advanced and expert CCE level of training. Monitoring of RV size and function or detection of fine abnormalities requires quantitative assessment.

## Additional files

Additional file 1: **Appendix 1.** Answer sheet for right ventricle (RV) size and function by subjective assessment. To be filled out by intensive care specialists or fellows with at least one qualification in advanced echocardiography. 2D echo images from 80 patients to be reviewed assessing RV size and function as normal, mild/moderately impaired, and severely impaired. **Appendix 3.** Contingency table used for sample size estimation based on possible agreement. **Appendix 4.** Agreement chart (Bangdiwala’s observer agreement chart) for binary data (normal vs abnormal) on right ventricle subjective size and function assessment. Please see the ‘Methods’ section or reference [21] for the description of interpretation if needed. **Appendix 5.** Agreement chart (Bangdiwala’s observer agreement chart) for categorical data (normal, mild/moderately impaired, severely impaired) for right ventricle subjective size and function assessment based on level of echo experience (less or more than 7 years). **Appendix 6.** Agreement chart (Bangdiwala’s observer agreement chart) for categorical data (normal, mild/moderately impaired, severely impaired) for right ventricle subjective size and function assessment based on participant view that they practised at level of cardiologist. **Appendix 7.** Agreement chart (Bangdiwala’s observer agreement chart) for categorical data (normal, mild/moderately impaired, severely impaired) for right ventricle subjective size and function assessment based on participant qualification (DDU vs other) (PDF 361 kb)

## Abbreviations

ASE: American society of echocardiography
CCE: Critical care echocardiography
CICM: College of Intensive Care Medicine of Australia and New Zealand
DDU: Diploma of diagnostic ultrasound
Echo: Echocardiography
EDA: End-diastolic area
FAC: Fractional area change
IQR: Interquartile range
RV: Right ventricle
RVfwS: Right ventricle free wall strain
S′: Systolic velocity on tissue Doppler imaging
SD: Standard deviation
STE: Speckle-tracking echocardiography
TAPSE: Tricuspid annular plane systolic excursion
USIG: Ultrasound Special Interest Group
