# Supplementary material for: Subjective right ventricle assessment by echo qualified intensive care specialists: assessing agreement with objective measures
Source: Crit Care. 2019 Mar 7;23:70. doi: 10.1186/s13054-019-2375-z (PMC6407213; doi:10.1186/s13054-019-2375-z)

Appendix 1

How many years have you been using echo? (Give single value, not range)	
How many TTE do you perform a year? (Give single value, not range)	
Do you perform echo at level of a cardiologist?	Yes / No
How many years have you been an ICU specialist? (Give single value, not range)	
What formal echo qualifications do you have?	

Please indicate ***single*** category for **RV size** and **function** for each case based on 'eyeballing' videos

**Case**	**RV size**			**RV function**		
	**Normal**	**Mild/ moderate**	**Severe**	**Normal**	**Mild/ moderate**	**Severe**
1						
2						
3						
4						
5						
6						
7						
8						
9						
10						
11						
12						
13						
14						
15						
16						
17						
18						
19						
20						
21						
22						
23						
24						
25						
26						
27						
28						
29						
30						
31						
32						
33						
34						
35						
36						

Case	RV size			RV function		
	Normal	Mild/ moderate	Severe	Normal	Mild/ moderate	Severe
37						
38						
39						
40						
41						
42						
43						
44						
45						
46						
47						
48						
49						
50						
51						
52						
53						
54						
55						
56						
57						
58						
59						
60						
61						
62						
63						
64						
65						
66						
67						
68						
69						
70						
71						
72						
73						
74						
75						
76						
77						
78						
79						
80						

Appendix 3

		Reference standard (RV free wall strain)			*Total*
		**Normal**	**Mild / moderate**	**Severe**	
**Subjective RV assessment**	**Normal**	12	4	0	16
	**Mild-moderate**	8	12	2	22
	**Severe**	0	4	8	12
Total		20	20	10	50

Appendix 4

RV size assessment

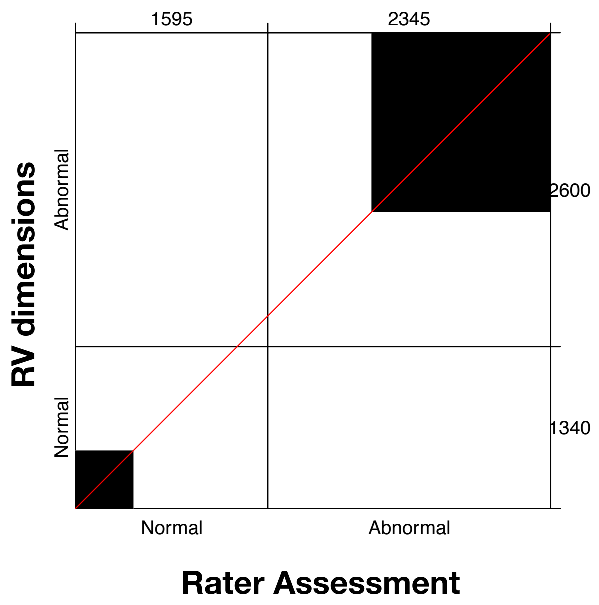

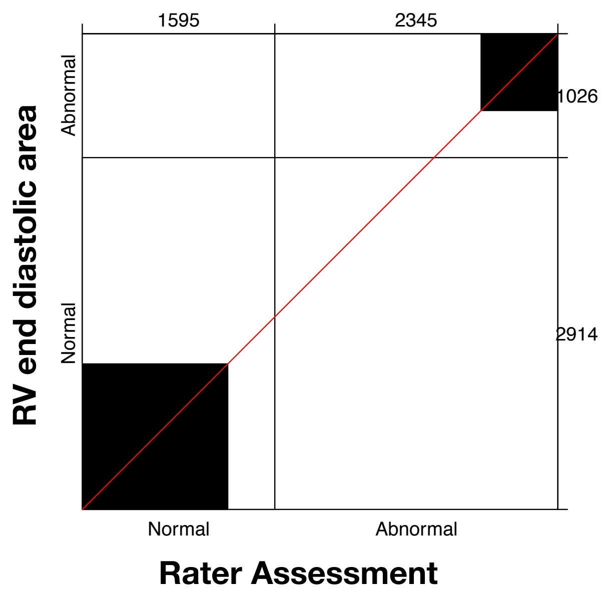

RV function assessment

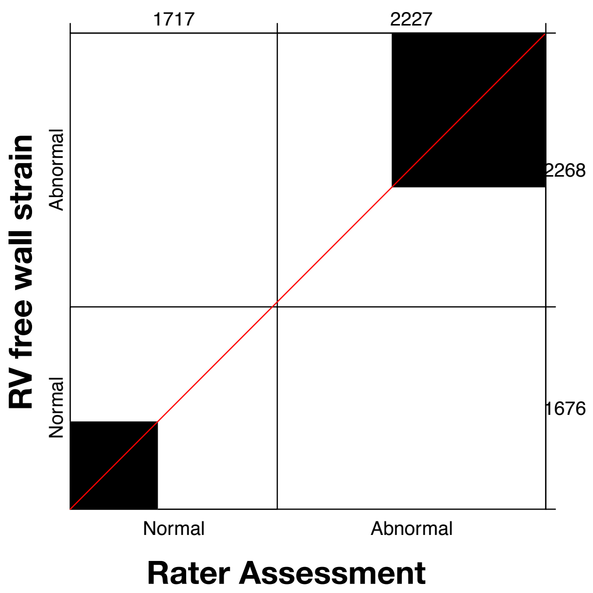

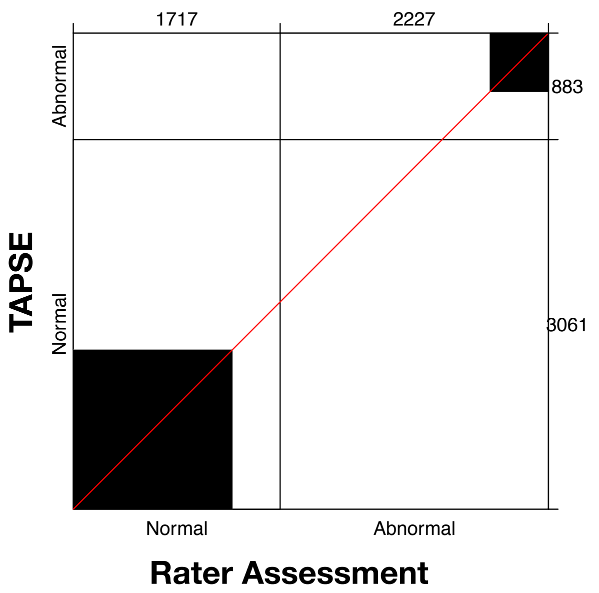

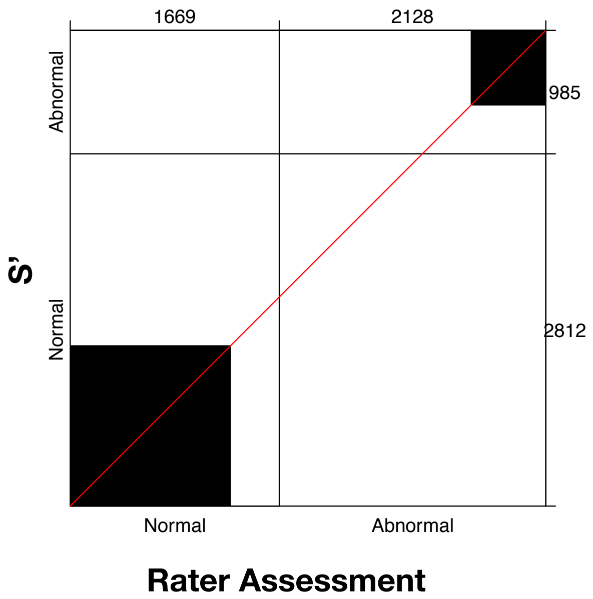

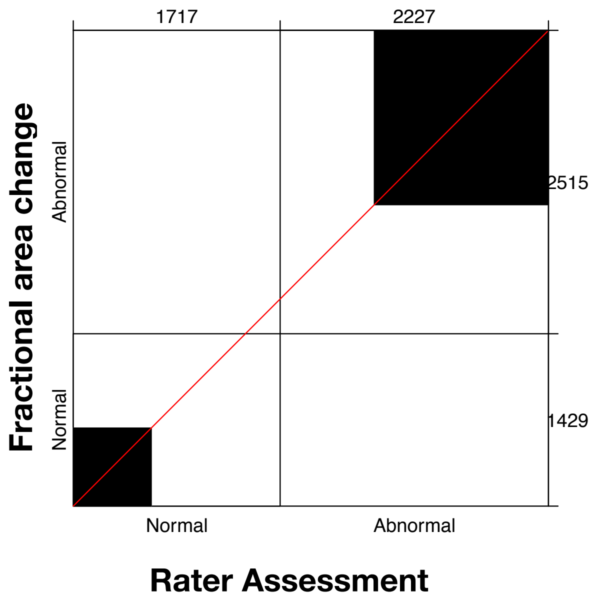

Appendix 5

Less than 7 years experience

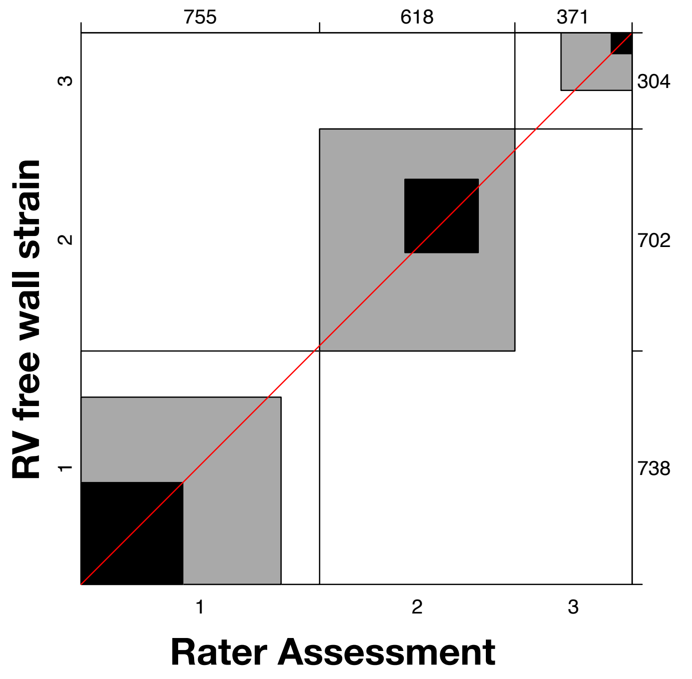

More than 7 years experience

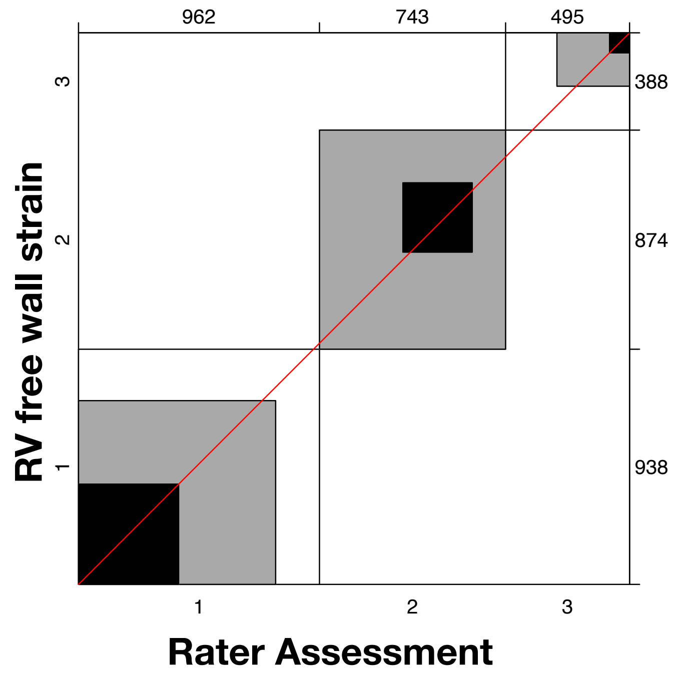

Less than 7 years experience

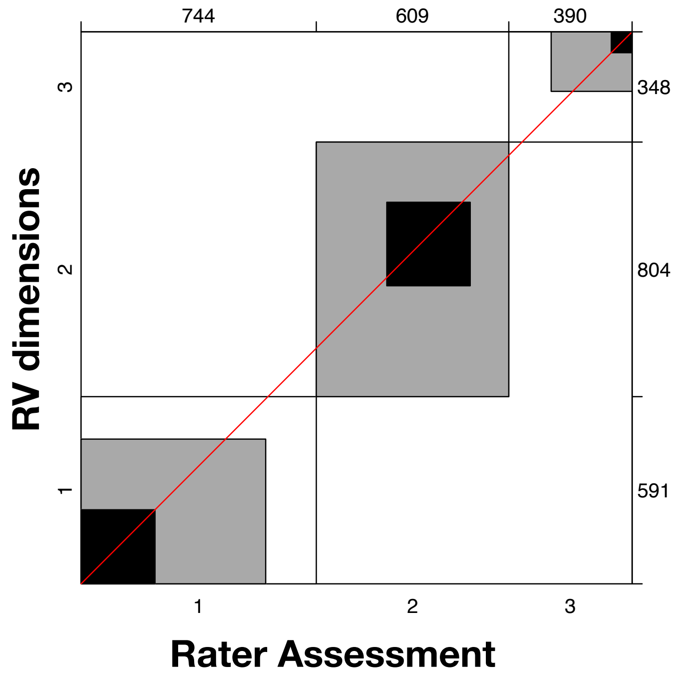

More than 7 years experience

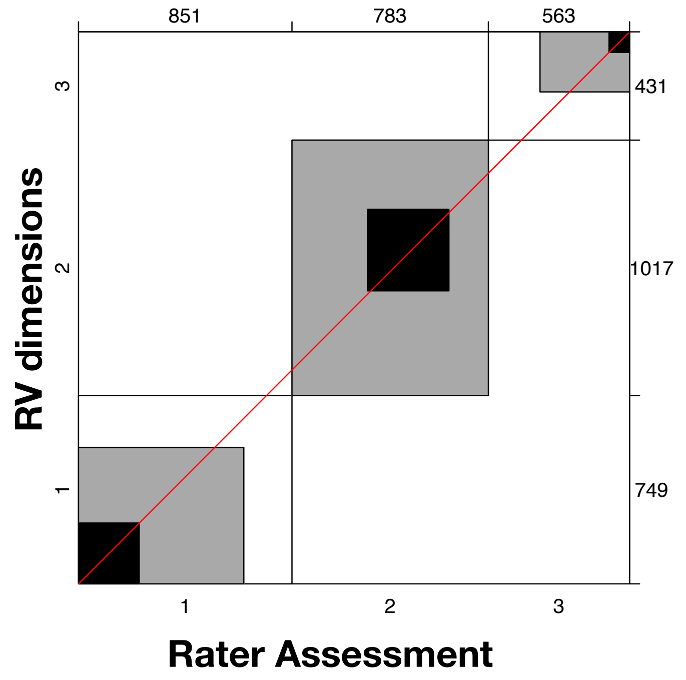

Appendix 6

Non-cardiologist

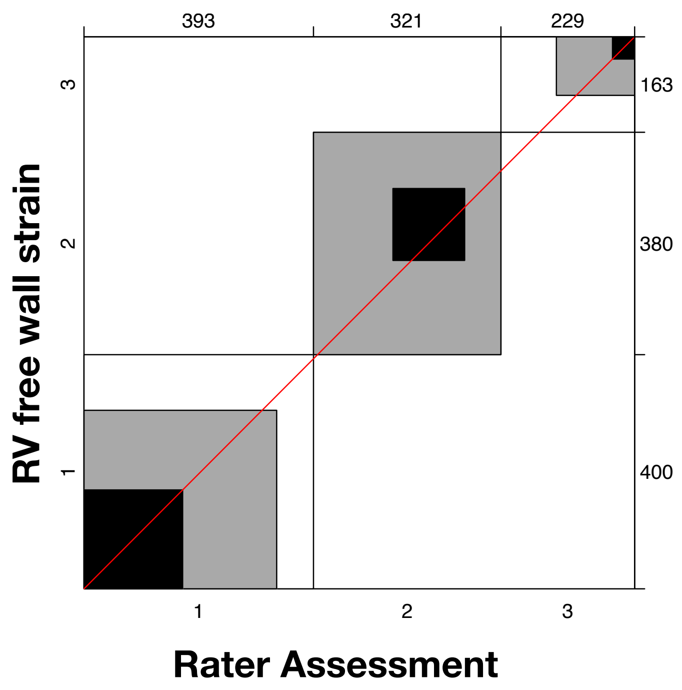

Cardiologist

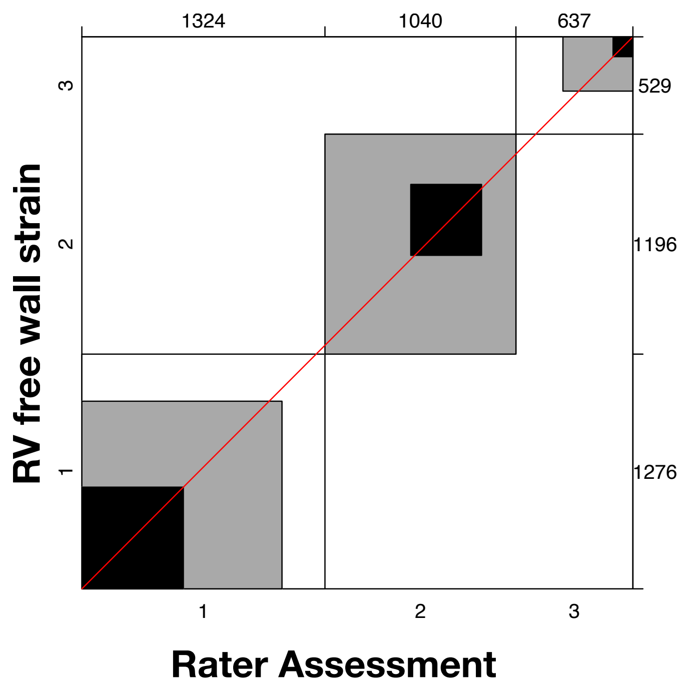

Non-cardiologist

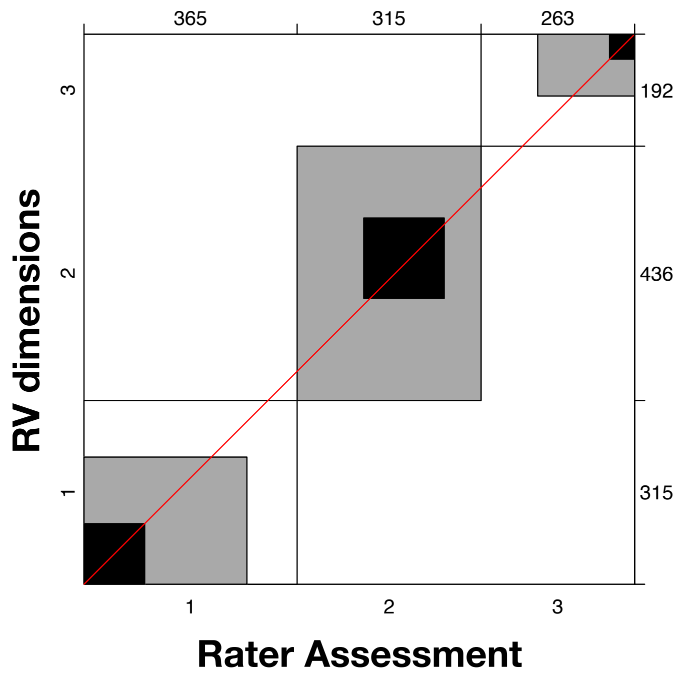

Cardiologist

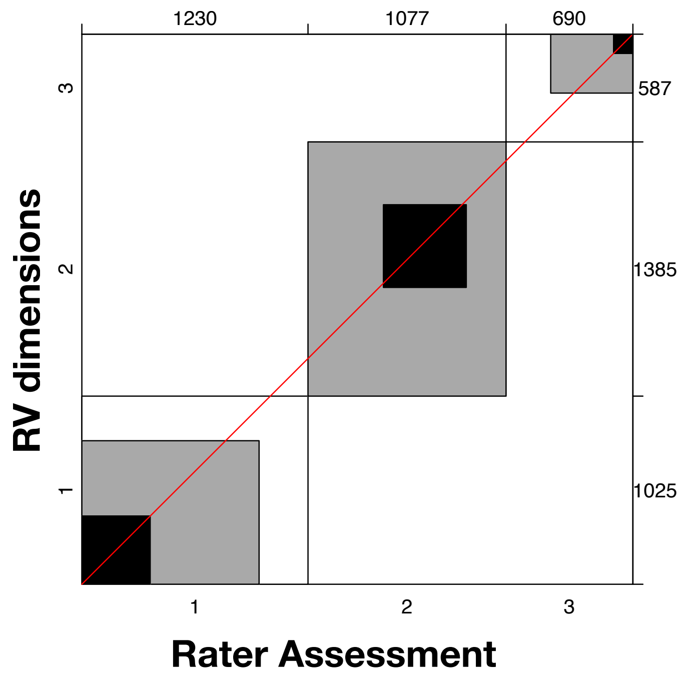

Appendix 7

Non-DDU qualification

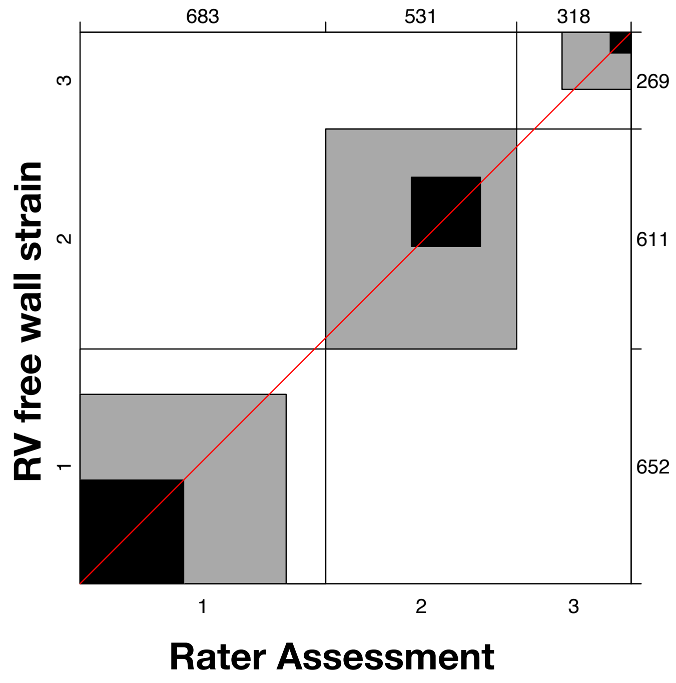

DDU qualification

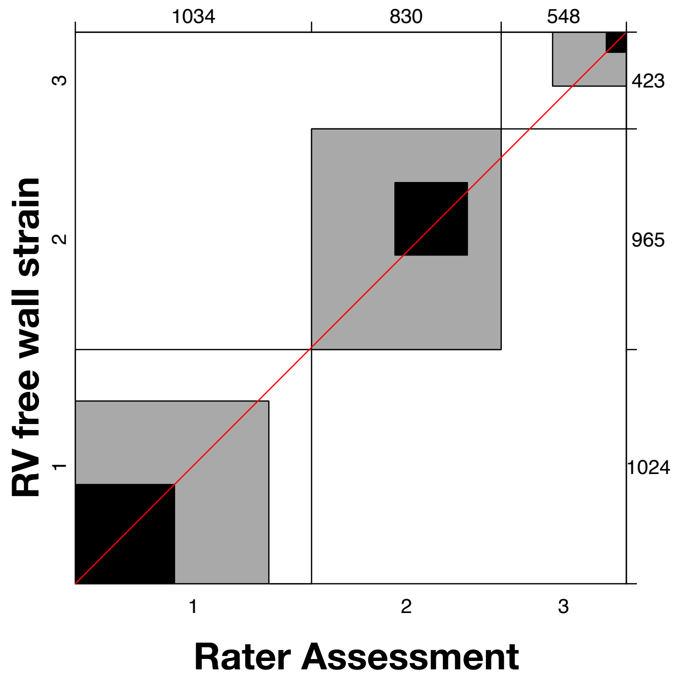

Non-DDU qualification

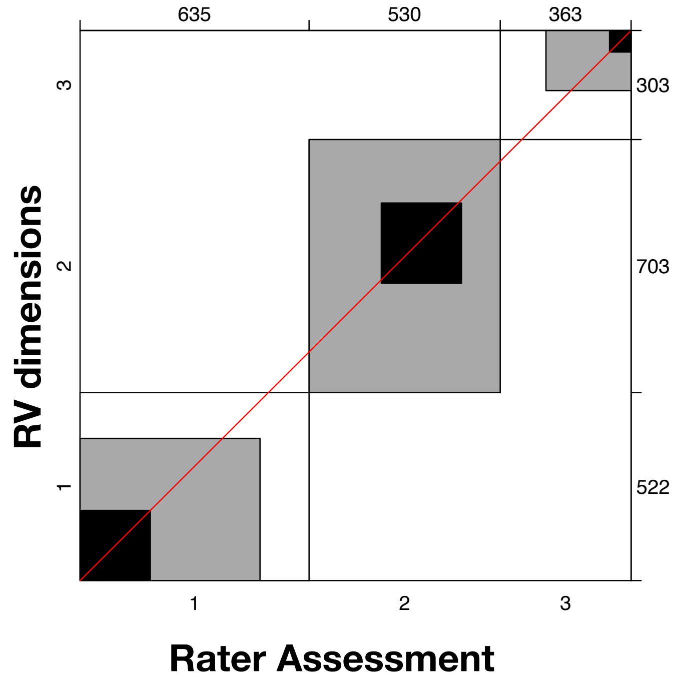

DDU qualification

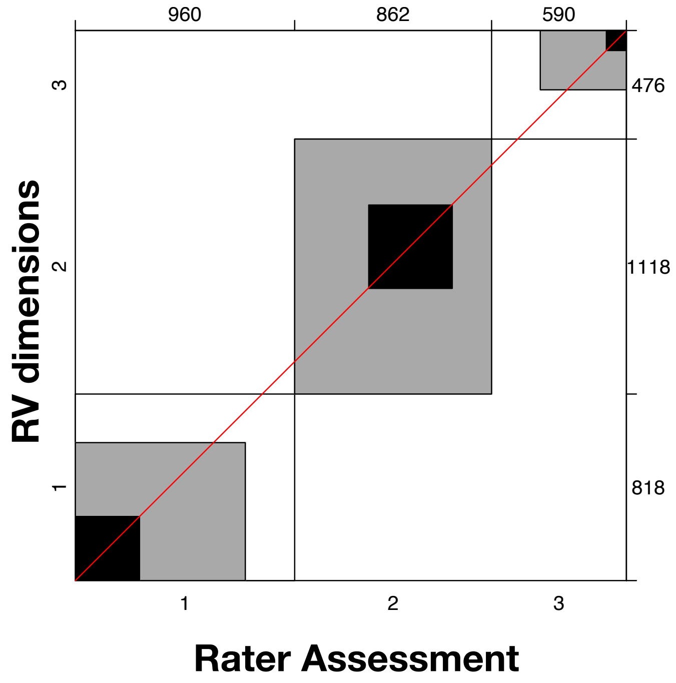

Supplement: Supplementary file 2 — Appendix 1. Answer sheet for right ventricle (RV) size and function by subjective assessment. To be filled out by intensive care specialists or fellows with at least one qualification in advanced echocardiography. 2D echo images from 80 patients to be reviewed assessing RV size and function as normal, mild/moderately impaired, and severely impaired. Appendix 3. Contingency table used for sample size estimation based on possible agreement. Appendix 4. Agreement chart (Bangdiwala’s observer agreement chart) for binary data (normal vs abnormal) on right ventricle subjective size and function assessment. Please see the ‘Methods’ section or reference [21] for the description of interpretation if needed. Appendix 5. Agreement chart (Bangdiwala’s observer agreement chart) for categorical data (normal, mild/moderately impaired, severely impaired) for right ventricle subjective size and function assessment based on level of echo experience (less or more than 7 years). Appendix 6. Agreement chart (Bangdiwala’s observer agreement chart) for categorical data (normal, mild/moderately impaired, severely impaired) for right ventricle subjective size and function assessment based on participant view that they practised at level of cardiologist. Appendix 7. Agreement chart (Bangdiwala’s observer agreement chart) for categorical data (normal, mild/moderately impaired, severely impaired) for right ventricle subjective size and function assessment based on participant qualification (DDU vs other) (PDF 361 kb) [file 13054_2019_2375_MOESM1_ESM.pdf]
